# What Does It Mean to Truly Empathize with a Patient? An Analysis of Japanese Cases Employing the Narrative Approach Perspective

**DOI:** 10.3390/healthcare10101884

**Published:** 2022-09-27

**Authors:** Akira Akabayashi, Eisuke Nakazawa

**Affiliations:** 1 Department of Biomedical Ethics, Faculty of Medicine, University of Tokyo, 7-3-1 Hongo, Bunkyo-ku, Tokyo 113-0033, Japan; 2 Division of Medical Ethics, School of Medicine, New York University, 227 East 30th Street, New York, NY 10016, USA

**Keywords:** empathize, village society, Fukushima, Great East Japan Earthquake, tsunami, Japan

## Abstract

What does it mean to truly empathize with a patient? The authors (a psychiatrist and a philosopher) explore this topic from the unique perspectives gained from decades of experience. We discuss how some approaches that may be criticized are necessary if we are to empathize with a patient. We also touch on the current situation surrounding personnel involved in the restoration of the Fukushima Daiichi Nuclear Power Plant (the so-called Fukushima 50) after the nuclear meltdown caused by the Great East Japan Earthquake in 2011. We conclude with what we have learned to truly emphasize with patients from these cases: (1) small deviations seem to be useful sometimes; (2) healthcare professionals do not have to be too hard on themselves to empathize with patients, and a natural, narrative approach-based attitude is sometimes more than enough; and (3) physical stance, in addition to psychological stance, might also be a factor. Moreover, we look at the idea of the “village society” and argue that it is necessary for medical professionals to be fully aware of the negative connotations of village societies.

## 1. Introduction

What does it mean for medical professionals to truly “empathize with their patients?” While a clear definition for this may be difficult to generate, empathy seems to be a common concept used readily in the healthcare field. For example, in nursing, we often hear that “patients require more attentive care” or “a medical professional must empathize with the patient to provide good medical care.” However, it is difficult to specify exactly what constitutes empathy or empathizing without specifying a particular context. We assume that in Japanese, sympathy is translated as *dojoh* and empathy as *kyokan.* However, the two are often used interchangeably outside of academic discussions within psychology. In the field of Japanese medicine, it is rarely said that medical professionals should “sympathize” or “empathize” with their patients. In fact, the colloquial expression often used is *yorisou.* In this study, we explore the question: “What does it mean to truly empathize (*yorisou*) with a patient?” We chose to focus on “empathy” because “*yorisou*” is not only about sympathy but also involves aspects of empathy. *Yorisou,* as we see it, has cognitive and affective domains, which are not very different from those of empathy, as described by Hojat [1].

Empathy has been described as a concept involving cognitive as well as affective or emotional domains. The cognitive domain of empathy involves the ability to understand another person’s inner experiences and feelings and the capability to view the outside world from the other person’s perspective. The affected domain involves the capacity to enter into or join the experiences and feelings of another person [1] (p. 1563).

However, *yorisou* has another meaning that is related to psychological proximity and physical proximity. The everyday use of *yorisou* sometimes includes an element of physical proximity, whether consciously intended or not. For example, if a child is crying in the distance, someone will say, “Go close to them and *yorisou*.” In addition, in a medical situation, a nurse at a terminally ill patient’s bedside at a hospice, holding their hand as they are about to breathe their last breath alone and without family or relatives, is probably doing much more *yorisou* (empathizing) than one drinking tea at the nurses’ station. In this study, we use “empathize” as the English translation of *yorisou*, but we believe it does not have exactly the same meaning, as described by Hojat [1].

Japan is a collectivist society, and has sometimes been called a “village society.” Historian Yusaku Matsuzawa says that “*mura-shakai*” (village society) means that human relations in modern Japan (for example, human relations in a company) are closed and conservative, placing importance on peer evaluation and on not changing customs. Consequently, it can become impossible to achieve the desired goal (e.g., to turn a profit, in the case of a company) [2]. Notably, the term “*mura-shakai*” does not necessarily refer to the local community. Rather, it refers to the general nature of communities that Japanese people tend to create. In a “*mura-shakai*”, homogeneity develops through mutual surveillance, but this is not necessarily uncomfortable for its members, as the resulting comfortable space is dominated by a system of mutual pampering [3]. Of course, the term “*mura-shakai*” has a negative nuance, as does the term “nuclear village”, [4] which was coined after the nuclear accident following the Great East Japan Earthquake to refer to the community of government, industry, and academic interests related to nuclear power plants that received a lot of negative press. 

In addition, village societies have been analyzed using the keywords “isolation”, “nature-based”, “peripheralization”, and “autonomization” [5]. “Isolation” refers to the separation of a society from other communities by geographical, physical, social, and psychological conditions [5]. When Japanese society is viewed as a village society, the focus is on Japan’s international isolation. Japan is an island nation that once adopted a strict isolation policy. Japan’s unique culture and values have created a gap between Japan and other countries, especially socially and psychologically, even in today’s globalized world [6]. This gap is not only between western countries and Japan but also exists within the Asian region. The term “nature-based” refers to the fact that society is built on the foundation of nature, in defiance of the modern value of controlling the natural environment. If we consider Japanese society as a whole as a village society, we can extract an aspect of Japan that is excessively hesitant to introduce rational systems. For example, Japan lags behind other countries in the rationalization of social systems through information technology [7]. “Peripheralization” refers to the reality that a local society is positioned on the periphery of a highly modernized society. Since it is positioned on the periphery, the local society only receives the products of the highly modernized society at its center with a time lag. If Japan as a whole is a village society, it is located in the Far East, just off to the east of the center of Asia, and is, therefore, peripheralized, falling into a state of simply following the products and social systems produced in the advanced regions of the world. “Autonomization” refers to the formation of a social system that is relatively independent of the national and local governments through voluntary self-governing organizational activities. Applied to Japan as a whole, it refers to the fact that traditional, often unstated, tacit customs are still deeply rooted in society, and failure to comply with such customs may result in exclusion from the community [8,9].

## 2. Methods

This paper employs case analyses. The first author, AA, is a male in his 60s whose specialty is psychiatry (as well as a part-time hospice physician) and medical ethics. The second author, EN, is a male in his 50s whose specialty is philosophy. During the case analyses, we have our sights set on narrative perspectives to explore what exactly it means for a healthcare provider to empathize with the patient. The discussion did not address one specific methodology of the narrative approach per se but refers to the common concepts underlying the narrative approach. Accordingly, the discussion will be written in the style of an essay.

## 3. Cases and Discussion

### 3.1. Part 1: The Case of a Young Female Patient

This case is from when the first author (AA) worked part-time at a mental health clinic.

#### Case

*At the end of 2018, seven years after the Great East Japan Earthquake, AA met with a female patient in her early 30s whose chief complaints were insomnia, anxiety, and mild depression (probably because of an adjustment disorder) and began to enter this in her chart. This was a completely routine task in a psychiatric examination room. He inquired about the complaints among the above symptoms and then asked about the course of her illness and about her medical history. Then, as he did with all patients, he asked, “If you don’t mind, would you tell me about the composition of your family?” It was then that it happened. The patient, who had been calm up until that point, suddenly panicked and began to sob uncontrollably. After about 15 min, she regained her composure and began to talk.*

*She first told AA that her hometown is in Iwate (Tohoku). She then recounted how the 2011 Great East Japan Earthquake and tsunami not only completely destroyed her house but swept her mother and younger female cousin out to sea. Her father is now struggling to manage his high blood pressure and diabetes, and her younger sister does not work but stays home to care for her ailing father. The mention of family by the physician during the examination had unintentionally evoked a flashback, triggering PTSD.*

*At a former workplace, her boss had told her “you are using the disaster as a crutch”. This was an incredibly insensitive thing to tell someone who lost family members in the tsunami. She modified her original complaint to note “I realize that my current workplace is somewhat better than my old one.”*

*She noted that she had no intentions of getting married in the future and was planning to return home to care for her family. She also said that if people discovered that she was an earthquake victim, she would be discriminated against and ostracized and that men would think twice before marrying her because of this.*

As explained above, being different from others in a village society often means that you will be labeled, perhaps shoulder some stigma, and likely be ostracized. In addition, people outside of your own village (community) are relatively less important. This patient’s boss, who said “you are using the disaster as a crutch”, was not concerned about her feelings because, although they worked at the same company, the patient was not a member of the boss’s immediate family or village. 

The victims are never the ones to blame for a disaster. However, victims of tragedies somehow become differentiated from non-victims and face isolation from the community; this is typical of village society behavior. The government has invested large amounts of public funding in epidemiological studies disguised as post-disaster health surveys but has not reached out to the many victims who still suffer from the aftereffects of the disaster.

Her narrative here is her “past” (she lost her family in the earthquake and tsunami), her “present” (she is being punished at her workplace by the village society), and her “future” (she is not married and is thinking of returning to her parents’ house to take care of her family). All AA could do as her doctor was to prescribe sleeping pills, anti-anxiety medications, and some antidepressants, and listen to her carefully (often labelled superficially as “attentive listening”). AA always respected what she said and listened to her without disagreeing. AA never responded with any paternalistic retorts such as “no, no, no, you are still young, you should get married, have children, and start a new happy family”, which would make her wounds more painful. For this patient living in this village society in Japan, would we conclude that AA treated her with empathy?

At her final visit with AA, she said that she was quitting her job and returning home to her parents’ house. It was at this point that AA expressed an opinion for the very first time with her, noting extreme disappointment in the Japanese village society and the lack of governmental effort to reach victims like this patient. In an effort to make her story known, he asked her if she would sign a consent form to allow him to tell her story anonymously in a publication. She smiled and was more than happy to sign it, and as a result, her story is presented publicly here for the first time. 

### 3.2. Part 2: Some Deviations 

The following episodes are from AA’s many years of clinical experience; these are conflated cases as the content has been significantly modified to protect patient anonymity.

(1) On transference cure

The following is a case that drastically changed AA’s view of psychotherapy. Please note that AA has several years of Freudian psychoanalytic training but has not undergone educational analysis.

*The patient, a middle-aged woman, came to the hospital with complaints of depression and suicidal thoughts and was prescribed antidepressants. She was highly intelligent and married. After roughly five years of treatment, she began to talk about her hobbies and seemed to have stabilized, but one day she asked “what is the ideal medical patient–doctor relationship? I understand that it is ideal to have appropriate distance and trust, which leads to better medical care. But shouldn’t the relationship be fluid as well? This is a field that deals with the human mind, isn’t it?”*

AA immediately felt that he had failed and was on the verge of a morbid positive transference. He re-contracted the patient to see her for 10 min per month and switched to a medication-only approach. Soon after, the patient told him “*when I was young, I was in pain because I could not stop my son from killing himself with a phone call to me right before he committed suicide. My son did not say that he wanted to die at all. I told you (AA) over and over again that I wanted to die.*” She continued, “*five years ago, when I first saw you, you told me ‘don’t die;’ you bent your pinky finger and asked me to ‘pinky swear’ that I wouldn’t give up on life. You smiled at me and said, ‘I need you to pinky swear that you won’t die.’ (Of course, AA never touched her, just bent his pinky finger from the desk.) That promise has kept me going for the past five years. That feeling isn’t a ‘transference’ of any kind whatsoever!*”

AA had some knowledge of psychoanalysis and was trained to “always maintain neutrality between doctor and patient” but was unaware that the mere act of bending one’s pinky finger could have had such a powerful influence. Other psychiatrists might criticize AA for being too close to the patient. However, without that pinky swear, the therapeutic relationship may not have lasted for five years, as she may have committed suicide.

This is a case commonly referred to as a “transference cure.” I would anticipate a great deal of criticism from doctors in the field of psychoanalysis, but the major lesson learned here is that empathizing with a patient is not necessarily something that one does consciously; rather, as demonstrated in this case, it is possible for one to empathize unconsciously. This may take the form of a casual or small gesture by healthcare professionals. It may be as simple as a nurse’s routine measurement of blood pressure. The gentle application of the blood pressure cuff may display a healthcare provider’s attentiveness to the patient. Medical professionals do not have to try to force empathy, but rather walk with a patient as they journey through their ailing period. Moreover, in this case, the psychological distance between the patient and doctor is very close. This deviation might influence doctors’ sympathetic attitude toward patients, although this comes with risks. This deviation has many pros and cons.

This particular patient had a sad past, a present in which she had managed to suppress her desire to die but with PTSD, and an uncertain future. Despite the potential for criticism from others, AA chose to stay with her a little more in the transference cure state, while also being very careful to avoid morbidly positive transference. 

(2) On the medical professional’s pretense of being a medical professional

Medical education curricula tend to emphasize the need to refrain from talking about oneself. Even in situations of psychoanalysis, if a client asks a therapist about the therapist’s own preferences, psychoanalysis tutors teach one to “interpret” and maintain neutrality, replying with other questions, such as “why do you ask that?” or “what is your concern?”

The next case, also a middle-aged woman, presented with many mental and physical complaints and was always frustrated and in a bad mood. She was taking Chinese herbal medicine and anti-anxiety medication as needed and was told that she was suffering from symptoms related to menopause. Her family comprised her husband and a son (a college student). They were financially stable.

*One day, she entered the doctor’s office with a grim expression on her face as usual. As soon as she entered, she launched into endless complaints about her son, who would not listen to anyone and in some cases was not returning home at night. At some point, AA stopped and shared his own experience with her. He said “Mrs. A, I have a son in college, too, and he doesn’t seem to acknowledge that I am here for him either. He never responds to my e-mails. However, twice a year I get a one-sentence e-mail from him. On what occasions would you think he sends these?” Mrs. A replied, “Is it when he needs you to send money or something?” AA replied, “Happy Birthday” and “Happy Father’s Day”. The patient’s face suddenly relaxed and she smiled broadly. “I thought it was just my son. It is the same with you, doctor! But why does your son remember your birthday?” AA answered “I have a trick. When I made him a credit card, the hint I gave him for the four-digit PIN was only that it was my birthday. My son has to remember my birthday every time he withdraws money.” Mrs. A and AA had a good laugh together, and Mrs. A left in a very good mood that day.*

It may be true that healthcare professionals should not talk about their private lives, but it may be more natural for them to talk about themselves occasionally, depending on the situation. This is because if the patient is not a psychiatric patient, the risk might be low, and nothing is more valuable than a trusting patient–healthcare professional relationship. If healthcare professionals are too obstinate, they may risk destroying the trust implied in the patient-physician relationship. 

This is another example of how finding common ground with the patient can be an effective way to get to know them.

### 3.3. Part 3: Fukushima 50

One topic we wanted to include in this report is the story of the Fukushima 50. While this does not look to relate directly to the theme of empathizing with patients, we would like to highlight and share an important aspect of the underlying story—a village society.

The academic aspects of the Fukushima 50 were covered in another paper [10], but we were unable to write about some of the controversies at that time. The Fukushima 50 were involved in the restoration of the Fukushima Daiichi Nuclear Power Plant after the meltdown of the plant following the Great East Japan Earthquake in 2011. These people were considered heroes at that time. However, as we wrote in our previous paper [10], their lives are now difficult. They receive no government subsidies to support their living expenses and do not even undergo a regular health survey. To make matters worse, they are being bullied by Japan’s village society. Evidently, if they mention at a job interview their involvement in the renovation of the Fukushima nuclear power plant, they will not be hired. If they say they worked in Fukushima, they face discrimination, and their children are often bullied at school. As such, some people cannot even tell their families that they worked at Fukushima Daiichi Nuclear Plant. Why can the government and the people not extend a helping hand to those who sacrificed so much for their country?

Past, hero; present, miserable; future, the same so long as they stay in the village society. This is the negative side of the village society. Anyone deviating from the norm finds themselves cut off from the herd as a result. 

Let us set aside the Fukushima 50 and return to the main topic of healthcare professionals’ empathy toward patients. We would assume that there are many patients who find it difficult, if not impossible, to talk about their past, who live their lives hiding their past, and who feel denigrated by the stigma of being ill or requiring treatment. In short, many people today are thought not to have anyone to talk to about their true feelings. Many people have forced themselves to suppress their feelings rather than express them; this is a common psychological defense mechanism. However, as has been demonstrated above, this defense is not impermeable and will often break down. Counseling can cost as much as JPY 10,000 (USD 80) per hour. Not many people can afford this. 

In order for medical professionals to provide the best possible care for their patients, they must first be fully aware of the negative aspects of this village society. They must listen carefully to what the patients want the medical staff to know about them, other than their illnesses, and not impose their opinions on them. Unless they are given ample opportunity to share all of their concerns with their caregivers, patients will remain in their shells for a long time. Many healthcare professionals may be tired of the slogan “the medicine must see the whole patient, not just the disease.” Without the embodiment of seeing the whole patient and being more attentive to the patient, these words simply ring hollow.

## 4. Conclusions

A Japanese psychiatrist and a philosopher analyzed some concrete examples—and explored the meaning—of “truly empathizing with patients” in an actual Japanese healthcare practice context. As observed above, Japan is a collective society (village society) and empathy (*yorisou*) in a village society includes: attentive listening; respect for patients as equal human beings; a trusting collaborative relationship, the same in the different types of society. However, by dealing with the cases of patients in Japan’s village society, we derived two learning insights: (1) occasionally, small deviations are seemingly useful. (2) Healthcare professionals need not be excessively hard on themselves to empathize with patients, and a natural, narrative approach-based attitude is generally sufficient. 

First, deviating from convention and societal expectations will not be appropriate in individualistic societies. However, in village societies—wherein people are more dependent on each other than in individualistic societies—healthcare professionals are expectedly depended on by the patients. In the first case of part 2, the psychological distance between the patient and doctor was slightly narrower, but the patient was, nevertheless, comfortable with this level of psychological distance. In the second case of part 2, the patient experienced relief when she came to know that she was not the only one and the same with others. In village societies, if someone is acting differently, one might be ostracized, as discussed earlier.

Secondly, we described earlier that healthcare professionals need not force themselves excessively hard to empathize with patients because in village societies, people are already dependent on each other to some extent. Accordingly, patients are not highly individualistic, and they would not ask healthcare professionals to deliver some form of special care that “suits” them. Especially in healthcare settings, patients’ natural assumptions and expectations are that healthcare professionals should empathize with the patients. Thus, in such a context, if a nurse diverts from her usual behavior when caring for a specific patient (e.g., spending more time listening to and staying with one patient, such as for more than 30 min), the patient could feel odd.

Following these remarks, we may conclude that the term “empathize” holds a common meaning worldwide, albeit it may present different components depending on the societies in which the term is used. This paper’s collective society’s use is a good comparison with individualistic societies.

Lastly, we intend to address a challenging concept: physical distance is also a relevant component of empathy, meaning that empathy does not encompass only the cognitive/affective perspective. We are well aware that this proposition greatly deviates from the current discussions in the psychology field. The only case we could introduce that shows the relevance of physical distance was what AA observed at the hospice (which can be checked in the Introduction Section), but we want to explore more regarding this challenging perspective in future research endeavors.

Furthermore, we would like to emphasize that although we discussed the negative aspects of Japan’s village society in the first Fukushima case, we do not intend to denigrate this society. Instead, we intend to underpin that every system has its natural advantages and disadvantages. Thus, although the village society may often be viewed negatively, it also has the advantage of promoting greater communal cohesion and richer human connections (as we explained above). However, we argue that medical professionals must be fully aware of the negative aspects of the Japanese village society.

Despite this paper having been written as an essay, which is a clear methodological limitation and that may be criticized as being non-scientific, we have tried to base our discussions on shared common concepts of narrative-based perspectives. Moreover, our goal in this study was keeping ourselves grounded and focused in real-world practices and settings while avoiding engagement in armchair discussions.

In sum, we deem that the concepts underlying the narrative approach are extremely useful and do broaden the healthcare professionals’ perspective in numerous ways. We hope that this paper helps readers explore and expand their own ideas regarding the acceptable meaning of a healthcare provider being “there” for the patient.

## Data Availability

Not applicable.
